# Biodegradable Metallic Glass for Stretchable Transient Electronics

**DOI:** 10.1002/advs.202004029

**Published:** 2021-03-15

**Authors:** Jae‐Young Bae, Eun‐Ji Gwak, Gyeong‐Seok Hwang, Hae Won Hwang, Dong‐Ju Lee, Jong‐Sung Lee, Young‐Chang Joo, Jeong‐Yun Sun, Sang Ho Jun, Myoung‐Ryul Ok, Ju‐Young Kim, Seung‐Kyun Kang

**Affiliations:** ^1^ Department of Materials Science and Engineering Seoul National University Seoul 08826 Republic of Korea; ^2^ Research Institute of Advanced Materials (RIAM) Seoul National University Seoul 08826 Republic of Korea; ^3^ Department of Materials Science and Engineering UNIST (Ulsan National Institute of Science and Technology) Ulsan 44919 Republic of Korea; ^4^ Biomaterials Research Center, Biomedical Research Division Korea Institute of Science and Technology (KIST) Seoul 02792 Republic of Korea; ^5^ Department of Oral and Maxillofacial Surgery Korea University Anam Hospital Seoul 02841 Republic of Korea; ^6^ Department of Nano Manufacturing Technology Korea Institute of Machinery & Materials (KIMM) Daejeon 34103 Republic of Korea

**Keywords:** amorphous alloys, biodegradable materials, metallic glass, stretchable electronics, transient electronics

## Abstract

Biodegradable electronics are disposable green devices whose constituents decompose into harmless byproducts, leaving no residual waste and minimally invasive medical implants requiring no removal surgery. Stretchable and flexible form factors are essential in biointegrated electronic applications for conformal integration with soft and expandable skins, tissues, and organs. Here a fully biodegradable MgZnCa metallic glass (MG) film is proposed for intrinsically stretchable electrodes with a high yield limit exploiting the advantages of amorphous phases with no crystalline defects. The irregular dissolution behavior of this amorphous alloy regarding electrical conductivity and morphology is investigated in aqueous solutions with different ion species. The MgZnCa MG nanofilm shows high elastic strain (≈2.6% in the nano‐tensile test) and offers enhanced stretchability (≈115% when combined with serpentine geometry). The fatigue resistance in repeatable stretching also improves owing to the wide range of the elastic strain limit. Electronic components including the capacitor, inductor, diode, and transistor using the MgZnCa MG electrode support its integrability to transient electronic devices. The biodegradable triboelectric nanogenerator of MgZnCa MG operates stably over 50 000 cycles and its fatigue resistant applications in mechanical energy harvesting are verified. In vitro cell toxicity and in vivo inflammation tests demonstrate the biocompatibility in biointegrated use.

## Graphical abstract

Fully biodegradable MgZnCa metallic glass (MG) film is proposed for intrinsically stretchable electrodes with a high yield limit (≈2.6%), exploiting the advantages of amorphous phases with no crystalline defects. The top image shows the highly stretchable MgZnCa MG electrode integrating serpentine structure. Dissolution images of fatigue‐resistant triboelectric nanogenerator demonstrate the application of intrinsically stretchable MgZnCa MG in repeatable stretching mode.

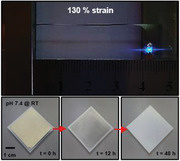

## 1 Introduction

Flexibility and excellent electrical performance and sophisticated fabrication can be ensured in inorganic materials, such as Si and metal nanomembranes, nanoribbons, nanomesh, and nanowires, if their thicknesses are made to be nanoscale.^[^ ^1^, ^2^, ^3^, ^4^ ^]^ Combining geometrical designs such as serpentine and mesh can further change the in‐plane to out‐of‐plane deformation, in nanoscale metal electrodes, thereby making stretchable electronics available.^[^ ^5^, ^6^, ^7^, ^8^ ^]^ This change in thickness imparts softness to the materials and also enhances their dissolubility in mild solutions such as groundwater, biofluids, and other aqueous solutions by tuning the time scale of electrochemical dissolution kinetics by a larger extent.^[^ ^9^ ^]^ Representatively, electronic‐grade Si of ≈100 nm thickness can completely dissolve in phosphate buffer saline (PBS) in roughly a month's span.^[^ ^10^ ^]^ Dissolubility of nanomaterials on a practical time scale opens up a new avenue for transient electronics that can fully dissolve in environmental fluids or biofluids in a controlled manner. Transient electronics provide unique features to biointegrated devices as they can be used both as wearable and implantable devices.^[^ ^9^, ^11^, ^12^, ^13^, ^14^ ^]^ Zero‐waste electronic patches are also available; in these patches, all the materials decompose into environmental and bio‐friendly materials after disposal.^[^ ^15^, ^16^, ^17^ ^]^ Minimally invasive implants and biodegradable electronic systems minimize surgical infections and complications by the elimination of the need for removal surgery; these are the other attractive applications of transient electronics.^[^ ^15^ ^]^

Stretchability is critically important for transient electronics in biointegrated applications to maintain the mechanical match between the electronic device and biological interface with the tissues, organs, and skin. Strain in biological systems typically ranges from 1% to 55% (for example, 1% strain is the deformation that arises from the artery pulse on the wrist, 55% strain arises in 135‐degree knee bending).^[^ ^18^, ^19^ ^]^ In addition, repeated stresses arise during daily movements and metabolic actions, and thus, understanding the fatigue behavior during stretching is essential. Transient inorganic conductive materials such as Mg, Zn, W, Mo, and highly doped Si have low elastic strain limits (less than 1%).^[^ ^20^ ^]^ Previous studies have proposed various strategies involving the use of island‐bridge structures to disperse the applied strain from serpentine‐shaped interconnects and 40% stretchability with Mg electrodes was demonstrated.^[^ ^11^ ^]^ However, the serpentine design provides material independent stretchability and limits the space available for electronic circuitry. Improving the elastic limit of transient metals provides intrinsic stretchability for space‐unlimited design and provides extremely enhanced stretchability combined with serpentine geometry.

Metallic glass (MG) is an attractive choice for the development of highly stretchable and fatigue resistive transient electrodes because of the following reasons. 1) It shows large elastic strains owing to its amorphous nature and lack of crystalline defects, such as, dislocation or grain boundaries, which are the source of deformation mechanism in crystalline materials,^[^ ^21^ ^]^ 2) individual element atoms of MG maintain their electrochemical behavior on dissolution in aqueous solution,^[^ ^22^, ^23^ ^]^ and 3) nanofilm formation is relatively easy via well‐studied fabrication processes such as, co‐sputtering.^[^ ^24^, ^25^, ^26^ ^]^ In particular, enhancement of elastic strains maximizes the repeatable stretching ranges as the plastic deformation accompanies the unrecoverable extension which distorts the devices. It also gives higher fatigue resistance, offering full recovery of deformation without damage accumulation.

Here, we propose a biodegradable electronic‐grade MG using an amorphous alloy of Mg, Zn, and Ca for the realization of stretchable and fatigue‐resistant transient electronics. The non‐uniform dissolution kinetics of MgZnCa MG was investigated in various aqueous solutions (deionized (DI) water, pH 7.0; PBS, pH 7.4; buffered solution (BS), pH 9.0) by through‐thickness X‐ray photoelectron spectroscopy (XPS). Nanomechanical tensile testing revealed that the yield strain and elongation of MgZnCa MG were higher than those of conventional transient nanofilm metals; additionally, improved stretchability and fatigue resistivity were demonstrated. Combined with the serpentine geometry, the MgZnCa MG interconnection enhanced stretchability by ≈1.5 times than that of Mg. The demonstration of transient passive and active devices, including a capacitor, inductor, PIN diode, and n‐type metal‐oxide semiconductor field‐effect transistor (MOSFET) showed the superior integration capability of MgZnCa MG compared to other representative transient electronic materials such as Si nanomembranes (Si NM), SiO_2_, and polybutylene terephthalate (PBAT) biodegradable elastomeric substrates. A transient triboelectric nanogenerator (TENG), which requires repeatable deformation with large strain, verifies the stretchability and fatigue resistance of the MgZnCa MG electrode. Finally, biocompatibility testing using in vitro cell toxicity and in vivo inflammation analysis supports the biofriendly usage of MgZnCa MG integrated electronic system.

## 2 Results and Discussion

### 2.1 Dissolution Chemistry and Kinetics

**Figure 1:**
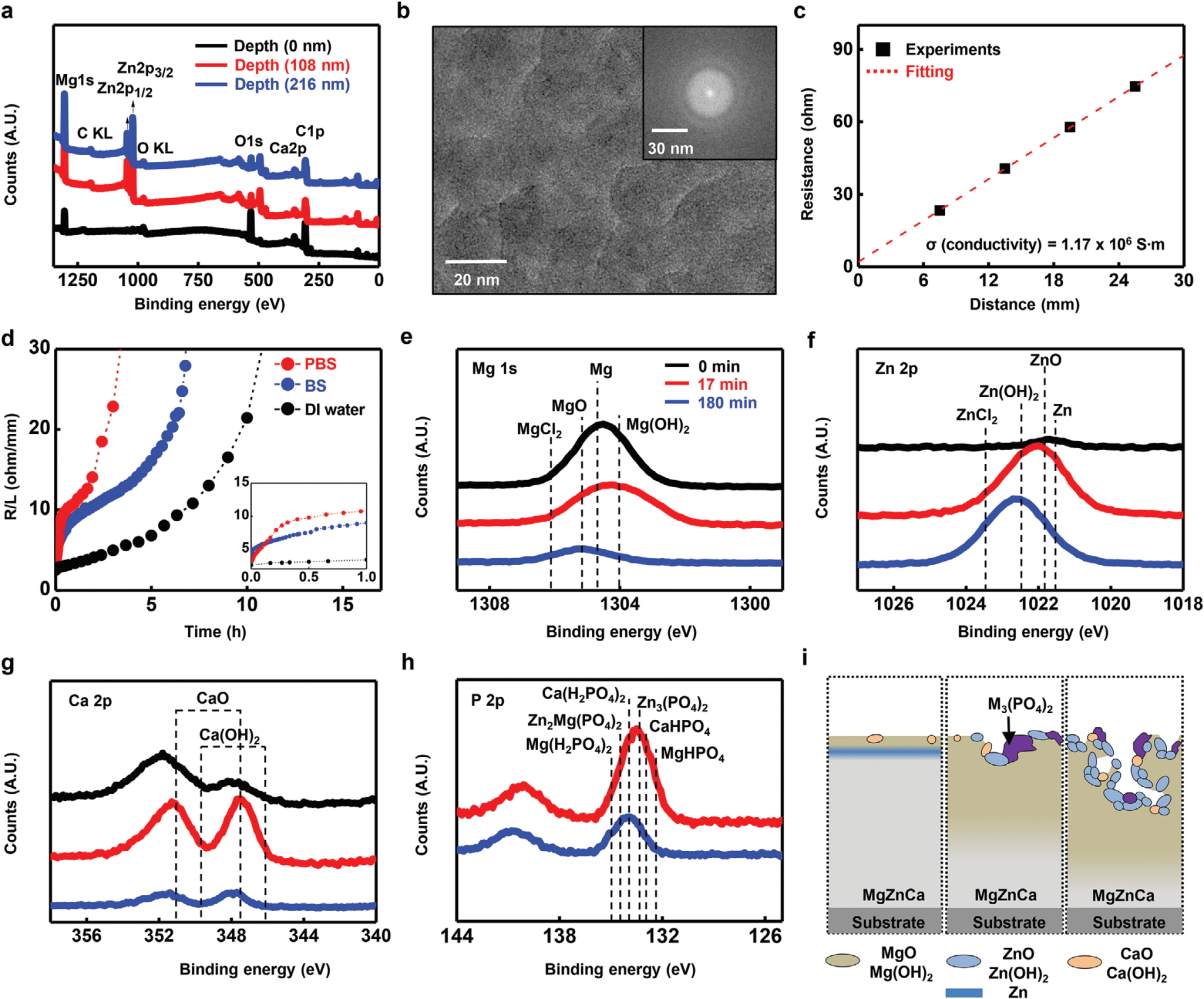
Dissolution chemistry and electrical property of Mg_67_Zn_28_Ca_5_ MG. a) Atomic composition of MgZnCa MG measured by XPS at different depths (black, surface; red, 108 nm; blue, 216 nm). b) Surface image and diffraction pattern (inset) observed by TEM. c) Two‐point resistance of MgZnCa MG resistor of thickness 200 nm, width 1.5 mm, and lengths from 7.5 to 22.5 mm. Conductivity calculated from resistor geometry was 1.17 × 10^6^ S∙m (resistivity 0.855 Ω µm^–1^). d) Transitional resistance change by non‐uniform dissolution of MG in PBS (pH 7.4), BS (pH 9.0) and DI water (pH 7.0) at 37 °C. Serpentine pattern has 300 nm thickness, 1.5 mm width and 82.5 mm length. Inset image shows magnified view, which time scale is 0 to 1 h. e–h) Change in surface chemistry demonstrated by binding energy of Mg 1s, Zn 2p, Ca 2p and P 2p for three dissolution periods (black, 0 min; red, 17 min; blue, 180 min) in PBS at 37 °C. i) Schematic image of dissolution process of MgZnCa MG immersed in PBS (left; 0 min, bottom; 17 min, right; 180 min).

**Figure** **1** shows the atomic structure, electrical properties, and dissolution behavior of the biodegradable Mg_67_Zn_28_Ca_5_ MG in electronic‐grade thin films. Co‐sputtering biodegradable Mg, Zn, and Ca formed amorphous alloys with relative amounts of 68.2 (±2.5), 27.1 (±2.3), and 4.7 (±0.8), respectively, as measured by electron dispersive X‐ray spectroscopy (EDS) (Figure S1, Supporting Information). XPS revealed the relative contents of Mg, Zn, and Ca at different depths with binding energy distributions (Figure 1a). Oxygen showed a higher intensity than Zn. Furthermore, Zn was found lower than at the depths of 108 and 216 nm. Mg and Ca formed native oxide layers, thereby inducing immediate Zn depletion at the outermost surface owing to exposure to oxygen and moisture in the ambient atmosphere, but the internal MgZnCa MG film has pure Zn in the matrix.^[^ ^27^ ^]^ The transmission electron microscopy (TEM) image and its fast Fourier transformation (FFT) – filtered electron diffraction pattern as seen in Figure 1b, which demonstrates the amorphous nature of the deposited films. Figure 1c shows the resistance variation in MgZnCa MG resistors formed on Si/SiO_2_ films with lengths ranging from 7.5 to 25.5 mm (width and thickness were 1.5 mm and 200 nm, respectively). The resistivity of the MgZnCa MG film was determined using the transmission line measurement by,

(1) $$\mathrm{Slope}=\rho /\left(w\cdot t\right)$$

where *ρ* is the resistivity and *w* and *t* are the width (1.5 mm) and thickness (200 nm) of the line patterns, respectively. The conductivity of MgZnCa MG is 1.17  ×  10^6^ S m^–1^ (resistivity was 0.855 Ω∙µm), which is comparable to that of non‐biodegradable MG films used in stretchable electronics, for example, CuZr‐based MG (≈5.6  ×  10^5^ to 10^6^ S m^–1^), and slightly smaller than that of conventional biodegradable metal, that is, Mg (2.3  ×  10^7^ S m^–1^).^[^ ^28^, ^29^ ^]^

Dissolution behavior and its effect on electrical properties are critically important in transient electronics applications. Individual metal elements and metal oxides degrade in PBS as per the following reactions.^[^ ^27^, ^30^ ^]^

(2) $$\mathrm{MO}+H_{2}O\to M{\left(\mathrm{OH}\right)}_{2}$$

(3) $$M{\left(\mathrm{OH}\right)}_{2}\;+2Cl^{-}\to \mathrm{MC}l_{2}+2OH^{-}$$

(4) $$M+2H_{2}O\to M^{2+}+2OH^{-}+H_{2}$$

(5) $$3M^{2+}+2P{O_{4}}^{3-}\to M_{3}{\left(PO_{4}\right)}_{2},\;\mathrm{where}\;M=\mathrm{Mg},\;\mathrm{Ca},\;\mathrm{Zn}$$

The dissolution rates of Mg, Ca, and Zn are different from each other and dependent on the ion type of the solution.^[^ ^31^ ^]^ The difference in the dissolution kinetics of individual elements causes the dissolution behavior in amorphous alloys to be non‐uniform; for example, amorphous indium‐gallium‐zinc oxide showed a non‐uniform topographical change owing to the different dissolution rates of In, Ga, and ZnO in the aqueous solution.^[^ ^32^ ^]^ Non‐uniform dissolution of MgZnCa MG led to a transition in the resistance variation (Figure 1d) and formation of island‐like morphology during dissolution (Figure S3, Supporting Information).

Figure 1d depicts the variation of the representative resistance normalized by the total length of the serpentine trace (Figure S2, Supporting Information, 1.5 mm wide × 67.5 mm long × 300 nm thick) in PBS (pH 7.4), buffer solution (BS) (pH 9.0), and DI water (pH 7.0) at 37 °C. Dissolution of thin films causes a change in the resistance, which can be represented as follows.

(6) $$R\;=\frac{R_{0}h_{0}}{h}\;$$

(7) $$h\;=h_{0}\;-v_{\mathrm{MG}}\cdot t$$

where *R, R*_0_, *h*, *h*_0_, and *ν*_MG_ are the resistance, initial resistance, thickness, initial thickness, and dissolution rate of the MgZnCa MG, respectively. The resistance variation of MgZnCa MG in PBS showed a transition and a large variation in the dissolution kinetics at 17 min. Figures 1e–h describe the XPS results of constituent elements on the outermost surface of MgZnCa MG films in PBS at 37 °C for 0, 17, and 180 min as well as a schematic of the chemical reactants during dissolution. The large amounts of Mg and Ca on the surface form oxides (MgO and CaO) and hydroxides (Mg(OH)_2_ and Ca(OH)_2_) via reaction (2) after 17 min (Figures 1e,g). Almost no Zn is initially (0 min) present at the surface due to the depletion behavior described, but large amounts of surface Zn and Zn(OH)_2_ are observed at 17 min after dissolution of the top native oxide layers of Mg and Ca (Figure 1f). XPS results suggest that large amounts of Mg and Ca are turned into non‐conductive metal oxides or hydroxides subsequently after 17 min during the first resistance transition, but some degree of conductivity is preserved due to the remaining Zn. Further immersion until 180 min after transition causes hydrolysis of the remaining Zn, which ultimately forms Zn hydroxides. XPS showed that Mg and Ca on the outermost surface were mostly hydrolyzed, and large amounts of Zn formed hydroxides at 180 min. The decrease in dissolution rate after the transition is expected due to the formation of dissolution reactants such as, hydroxide and phosphate which precipitates and acts as a protective layer (as per the reactions (2) and (5)) between metallic ions (Mg^2+^, Ca^2+^, and Zn^2+^) and ions in solution (PO_4_^3–^, HPO_4_^2–^).^[^ ^27^, ^33^ ^]^ The XPS results in Figure 1h shows the formation of metal phosphate on the surface of MgZnCa MG. A schematic image of the dissolution reactants on the surface of MgZnCa MG at 0 min (left), 17 min (middle), and 180 min (right) are demonstrated in Figure 1i, and their detailed material profiles are shown in Figure S4, Supporting Information. Irregular dissolution of MgZnCa MG was also observed in the morphology. Figures S3a,b, Supporting Information shows optical and scanning electron microscope (SEM) images of MgZnCa MG dissolved in PBS at 37 °C. The fast reaction of Mg and Ca (with higher reactivity than Zn) causes an island‐like non‐uniform dissolution surface on the MgZnCa MG film. A large amount of island‐like Zn is observed at the surface near the pattern edge (green arrow in Figure S3a, Supporting Information) at 17 min, and dissolution reactants such as metal chloride and phosphates appear (green arrow in Figure S3b, Supporting Information) at 180 min. The low edge definition and roughness of the serpentine patterned film using a shadow mask causes a faster reaction in the surface region and so, a phosphate precipitate region forms preferentially near the edge.

**Figure 2:**
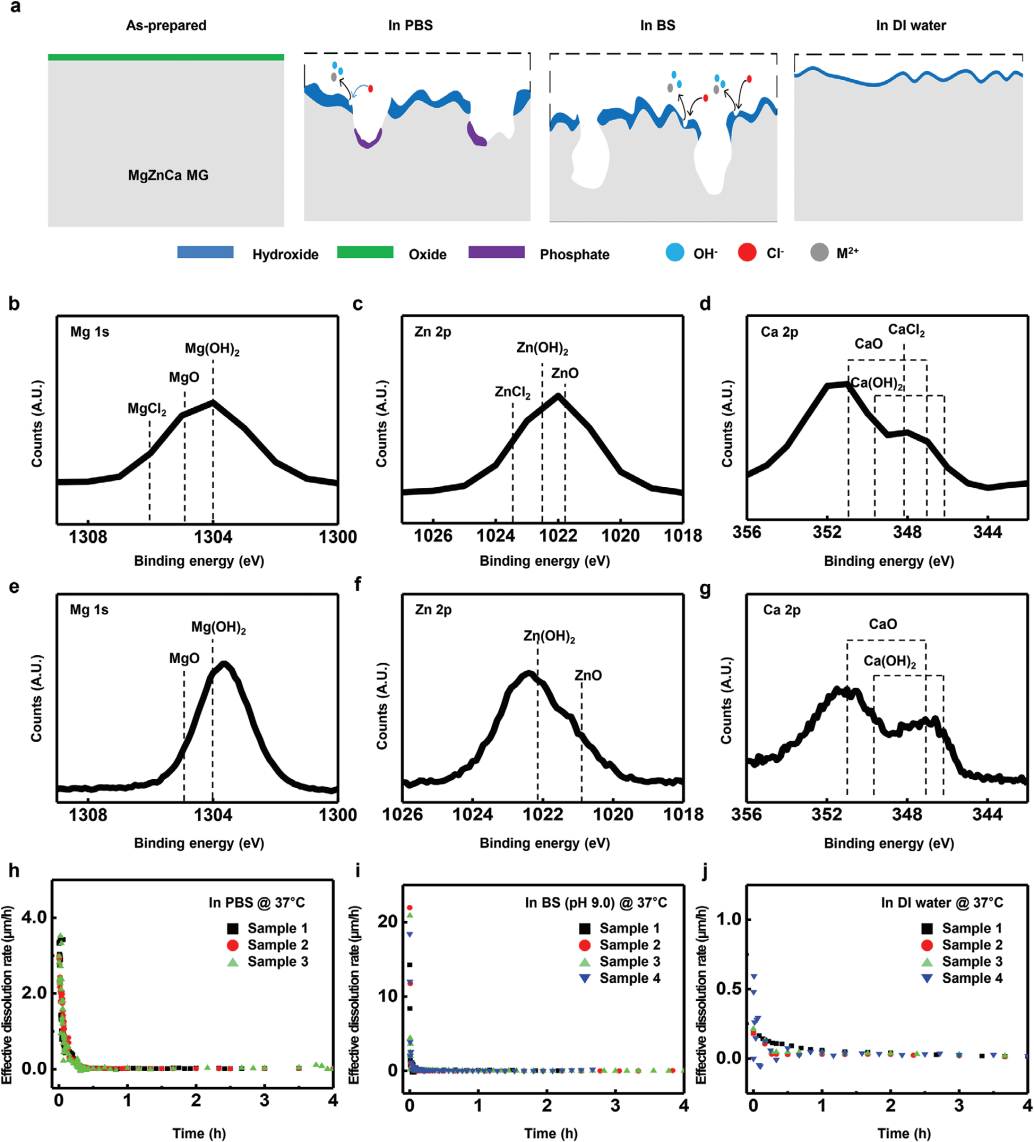
Dissolution chemistry depending on ion species in solution. a) Schematic diagram of surface evolution by ion species in DI water (pH 7.0), BS (pH 9.0), and PBS (pH 7.4). XPS analysis of b) Mg 1s, c) Zn 2p, and d) Ca 2p in BS (pH 9.0) immersion after 3 min. XPS analysis of e) Mg 1s, f) Zn 2p, and g) Ca 2p in DI water (pH 7.0) immersion after 30 min. Change in effective dissolution rate during immersion in h) PBS (pH 7.4); i) BS (pH 9.0); and j) DI water at 37 °C.

Dissolution kinetics also depends on the type of solution. **Figure** **2** represents the chemical product according to solution type (BS and DI water). Figure 2a shows a schematic of the formation of intermediate product during dissolution in three different solutions (DI, BS, and PBS) with different ion species. Cl^–^ ions accelerate the dissolution process by replacing metal hydroxides having lower solubility to metal chlorides with higher solubility.^[^ ^27^, ^33^ ^]^ The XPS results in Figure 2b–d show that metal chlorides exist on the surface of MgZnCa MG as per reaction (3), when MgZnCa MG immersed in BS. This phenomenon is also shown at immersion of MgZnCa MG in PBS (XPS results in Figure 1e,f and EDS data in Figure S2b, Supporting Information). MgZnCa MG in an ionic‐free solution, DI water, only shows hydrolysis as per the Equations (2) and (4). The XPS results in Figure 2e–g shows that the metal hydroxides formed as given in Equation (2) remain on the outermost surface protecting the inner MgZnCa MG matrix during dissolution in DI water. Figure 2h–j shows the effective dissolution rate of MgZnCa MG in three different solutions. Effective dissolution rate is calculated from Figure 1d using

(8) $$\frac{dh\left(t\right)}{dt}=R_{0}\;h_{0}\frac{d\left(\frac{1}{R}\right)}{dt}$$

The initial effective dissolution rates of PBS, BS, and DI water were 3.5, 20, and 0.4 µm h^–1^, respectively. Those effective dissolution rates decrease rapidly due to the fast chemical reaction of Mg and Ca. The initial effective dissolution rate in DI water is the slowest without transition. The dissolution rates in PBS and BS are faster than in DI water because of the presence of Cl^–^ ions. The initial effective dissolution rate of PBS is slower than that of BS at the early stage of dissolution due to the effect of phosphate precipitates.^[^ ^27^, ^33^ ^]^ The average dissolution rate during 3 h of PBS (≈0.73 µm h^–1^), however, is faster than that of BS (≈0.63 µm h^–1^) despite the presence of PO_4_^3–^. It is believed that this phenomenon is caused by the higher concentration of Cl^–^ ions (PBS; ≈140 mmol L^–1^) in PBS than in BS, which can offset the deceleration of dissolution despite the presence of PO_4_^3–^ ions (≈10 mmol L^–1^) in PBS.

### 2.2 Stretchability and Fatigue Resistivity

**Figure 3:**
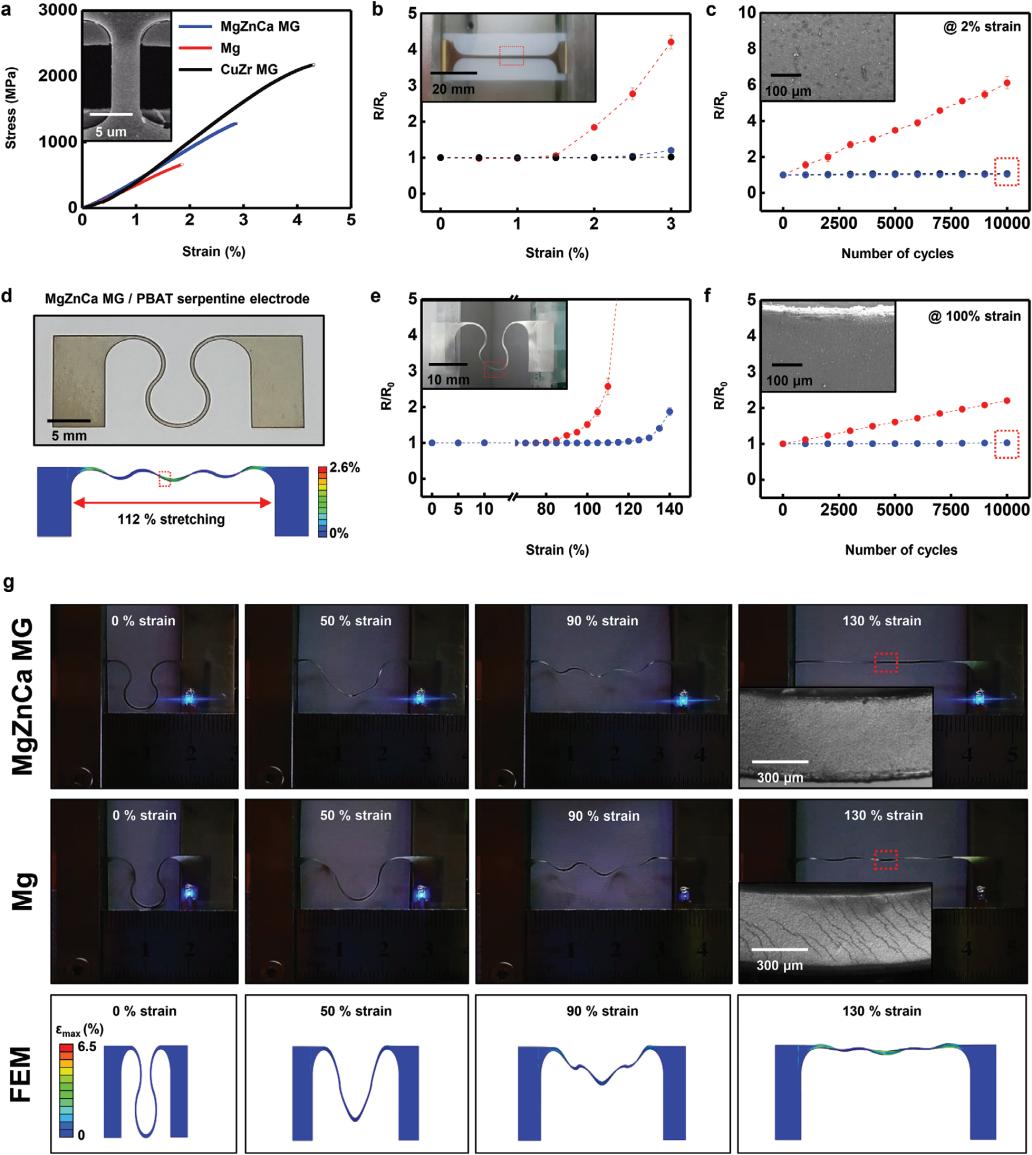
Uniaxial tensile and fatigue behavior of Mg_67_Zn_28_Ca_5_ MG. a) Stress–strain curve of free‐standing MgZnCa MG (300 nm thick, blue), Mg (300 nm thick, red), and CuZr MG (300 nm thick, black) films measured by nano‐tensile test. Inset: SEM image of dog‐bone‐type nano‐tensile sample of MgZnCa MG, 4 µm × 8 µm. b) Change in electrical resistance of MgZnCa MG (300 nm thick, blue), Mg (300 nm thick, red) and CuZr MG (300 nm thick, black) on PBAT substrate (≈150 µm) up to 3% tensile strain. Inset: image of sample, 1 mm wide and 40 mm long. c) Fatigue properties of MgZnCa MG (300 nm thick, blue), Mg (300 nm thick, red) and CuZr MG (300 nm thick, black) up to 10 000 cycles at 2% strain. Inset: optical image of MgZnCa MG after 10 000 fatigue cycles without cracking. d) Image of MgZnCa MG electrode (300 nm thick) deposited on PBAT substrate (150 µm thick) with geometrically enhanced stretchability. e) Resistance variation of serpentine MgZnCa MG (300 nm thick, blue) and Mg (300 nm thick, red) on PBAT substrate (≈150 µm thick) at strain up to 140%. f) Fatigue properties of serpentine MgZnCa MG (300 nm thick, blue) and Mg (300 nm thick, red) after 10 000 cycles at 90% strain. Inset: optical image of MgZnCa MG after 10 000 fatigue cycles without cracking. g) Stretchable serpentine electrode (top: MgZnCa MG, middle: Mg, bottom: FEM analysis of MgZnCa MG, Inset: OM image at strain concentration site) integrated with light‐emitting diode (LED) during uniaxial stretching up to 130%. LED of MgZnCa MG electrode shows consistent brightness without crack formation of MgZnCa MG, while one of Mg electrode is dimming after 90% strain with crack propagation. FEM analysis series shows strain distribution at 0%, 50%, 90%, and 130% strain.

MGs have superior mechanical properties of high strength and high elastic limit owing to their disordered amorphous structure.^[^ ^31^, ^34^ ^]^ A high elastic limit can enhance the stretchability and/or fatigue resistivity of MG electrodes. **Figure** **3** shows the mechanical characteristics of a nanoscale MgZnCa MG compared to crystalline Mg, which is the major element metal (68% ratio on average) of MgZnCa MG and is widely used in transient electronic devices.^[^ ^9^, ^11^, ^13^, ^14^ ^]^ In situ micro‐tensile tests were performed with a customized push‐to‐pull device using an in situ nanoindenter (Video S1, Supporting Information). Figure 3a shows the stress–strain curves of free‐standing MgZnCa MG, Mg, and CuZr MG fabricated into a micro‐tensile sample (8 µm length × 4 µm width, inset). Each stress–strain curve show linear elasticity and negligible plasticity, typical stress‐strain behavior. The elastic modulus, yield strain, and fracture strain of MgZnCa MG are 49.0 (±5.8) GPa, 2.6 (±0.6)%, and 2.9 (±0.4)%, respectively and those of Mg and CuZr MG are 36.8 (±4.8) GPa and 66.1 (±4.7) GPa, 1.5 (±0.2)% and 3.2 (±0.2)% strain, 2.2 (±0.2)%, and 4.2 (±0.1)% strain, respectively, in the same order. Here, the fracture strain of MgZnCa MG is larger than that of Mg although smaller than that of non‐biodegradable CuZr MG. MG generally show low plasticity because they lack plastic deformation mechanisms, while they generally have very high elastic limits because they combine high yield strength and low elastic modulus.^[^ ^21^, ^35^ ^]^ Mg generally shows low plasticity due to its hexagonal closed packed (HCP) crystalline structure. In particular, the Mg films in this study show brittle fracture because of their fiber texture and nanoscale grain size by which HCP plastic deformation mechanisms such as dislocation slip and twinning are suppressed.^[^ ^36^, ^37^ ^]^ Here the nanoscale MG showed greater elongation as well as elastic limit than the Mg films.

Figure 3b shows the variation in electrical resistance during uniaxial stretching of dog‐bone‐shaped MgZnCa MG (300 nm thick), crystalline Mg (300 nm thick), and CuZr MG (300 nm thick) samples on the PBAT (150 µm thick) substrate up to 3% strain. The resistance of the MgZnCa MG and CuZr MG electrode did not change up to 2% strain and increased slightly (≈1.2 and 1.02 times respectively) at 3% strain, while crystalline Mg electrode did not change up to 1% strain and increased by ≈4.2 times at 3% strain. In both MgZnCa MG and Mg, the resistance starts to increase below the yield strain (2.6% and 1.5%) because higher local strain occurs due to the stress concentration at the neck of the dog‐bone metal/polymer structure. The FEM analysis of the dog‐bone MgZnCa MG on the PBAT substrate in Figure S5, Supporting Information shows stress concentration at the sample neck due to the constraint at the bottom surface by the substrate and the free top surface. The local strain at the neck reaches a yield strain (≈2.6% and ≈1.5%) when the strain in MgZnCa MG and Mg samples reached ≈2% and ≈1.1%, respectively. In addition, MgZnCa MG and crystalline Mg electrodes show no electrical failure even at the fracture strain. Because the PBAT substrate can reduce the space needed for local elongation of a laminated metal film, it can suppress the local failure in the deposited metal film well beyond the fracture strain.^[^ ^38^ ^]^

MgZnCa MG shows better stability in cyclic deformation at large strain ranges owing to its greater yield strain and elongation than Mg. Figure 3c shows the fatigue resistivity (as described by electrical resistance) under cyclic deformation of MgZnCa MG, crystalline Mg, and CuZr MG electrodes on PBAT substrates during 10 000‐cycle stretching to 2% strain. The resistance of MgZnCa MG and CuZr MG remains stable in the 10 000‐cycle test, while that of Mg increases by about six times due to plastic deformation and local failure of crystalline Mg above its yield strain. The SEM image at the center of the specimen after 10 000 cycles also shows crack formation only in the Mg electrode (Figure S6, Supporting Information).

Structural interconnects using serpentine or mesh designs impart stretchability beyond the yield strain of the interconnect materials by converting in‐plane deformations to out‐of‐plane deformations.^[^ ^5^, ^6^, ^7^, ^8^ ^]^ Here, the increased yield strain of the interconnect materials dramatically enhances the stretchability of out‐of‐plane deformed structures. Figures 3d–g shows a strategy to enhance the electronic stretchability by combining the serpentine structure and high‐yield‐strain MgZnCa MG interconnections. A MgZnCa MG (≈300 nm thick) electrode on PBAT (≈150 µm thick) substrate was patterned into a serpentine structure (Figure 3d) by laser cutting. Figure 3e shows the variation in electrical resistance in serpentine‐structured MgZnCa MG/PBAT and Mg/PBAT during uniaxial stretching. Resistance is stable up to 115% of the stretch of the MgZnCa MG electrode and doubles at 140% stretch, while the resistance of the Mg electrode is stable only up to 75% stretch and shows an electrical failure beyond 130% stretch.

Applying a local strain to serpentine structure was studied by finite element analysis (Bottom of Figures 3d and Figures 3g). Serpentine‐structured MgZnCa MG offers up to a 30% increase in elastic stretchability over crystalline Mg with the same serpentine structure when the yield strain of MgZnCa MG is ≈1% greater than that of in crystalline Mg. The elastic stretchability in Figure 3e is comparable to the predicted FEA simulation with the yield strain of freestanding MgZnCa MG and Mg measured shown in Figure 3a. Figure 3g uses an operating LED to show the enhanced stretchability of the MgZnCa MG serpentine electrode compared to Mg. The LED with MgZnCa MG as a stretchable interconnect is stable up to 130% (130% is greater than the elastic stretchability, but it still has resistance (≈760 Ω) to operate the LED), while the Mg LED dims at 90% and fully extinguishes at 130%, showing a result comparable to Figure 3e. Series of FEM analyses in Figure 3g of the MgZnCa MG serpentine electrode at 50%, 90%, and 130% strain, respectively. Cyclic stretchability is also enhanced by the serpentine structure. Figure 3f shows the cyclic stretching behavior of serpentine‐structured MgZnCa/PBAT and Mg/PBAT after 10 000 cycles at 100% stretching: the serpentine‐ structured MgZnCa/PBAT shows negligible change in resistance after 10 000 cyclic stretches, while crystalline Mg increases by ≈2.2 times after the same cyclic stretches.

### 2.3 Transient Electronic Devices Using MgZnCa MG

**Figure 4:**
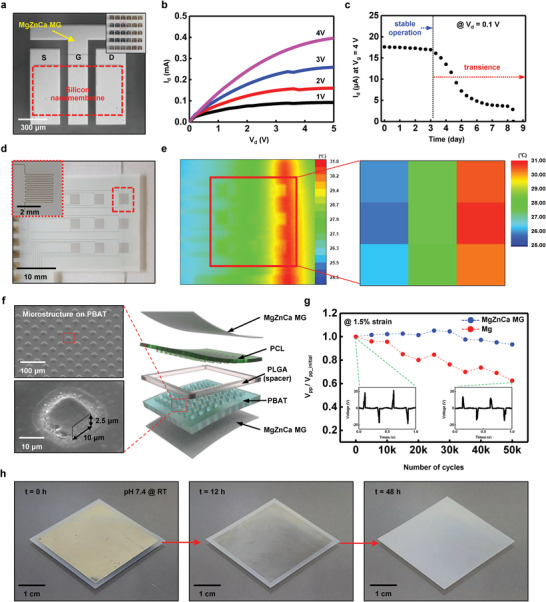
Electronics integrability of Mg_67_Zn_28_Ca_5_ MG. a) Image of biodegradable N‐MOSFET arrays made of Si NM active channel (≈400 nm), SiO_2_ gate oxide (≈100 nm), and MgZnCa MG electrode (≈300 nm). Channel length and width are 50 and 600 µm, respectively. b) *I*–*V* characteristics of N‐MOSFET. Mobility (linear region) and on/off ratio are ≈320 cm^2^ s V^–1^ and ≈10^5^, respectively. c) Transience of N‐MOSFET encapsulated by PBAT (≈100 µm). N‐MOSFET operated stably for 3 days in PBS at room temperature but lost its electrical performance over 6 days. d) Image of 3 × 3 array of MgZnCa MG temperature sensor made of 300 nm thick and 80 µm wide serpentine electrode traces. e) Temperature gradient measured by infrared camera (left) and transient temperature sensor array (right) on a joule‐heated rod. f) Exploded view of biodegradable TENG with top/bottom electrode (MgZnCa MG ≈ 100 nm thickness), spacer (PLGA ≈ 80 µm thickness), and micropatterned triboelectric layer (PBAT and PCL ≈ 100 µm) (right). SEM image of micropillar arrays of PBAT and PCL surface in top view (left). g) Fatigue degradation of output peak to peak voltage of TENG by cyclic bending with 1.5% maximum strain for MgZnCa MG (100 nm, blue) and Mg (≈100 nm, red) electrode. After 50 000 bending cycles, maximum output voltage of TENG of Mg electrode (inset) decreases by ≈40% compared to its initial state, while TENG of MgZnCa MG electrode shows negligible change. h) Series image of dissolution of MgZnCa MG TENG in PBS (pH 7.4) at room temperature for 48 h.

**Figure** **4** shows how MgZnCa MG can be integrated into transient electronic devices as a conducting layer. Figure 4a shows an optical microscopic image of n‐ channel MOSFET (called as N‐MOSFET) made of a ≈400 nm thick Si NM active layer (50 µm channel length and 600 µm channel width), ≈100 nm thick SiO_2_ gate dielectric, and ≈300 nm thick MgZnCa MG for the source, drain, and gate electrodes. The inset image shows arrays of N‐MOSFET on a glass substrate (see the fabrication details in the Experimental Section). Figure 4b shows the current‐voltage characteristics of an N‐MOSFET where mobilities and on/off ratios are ≈320 cm^2^ s V^–1^ and ≈10^5^, respectively (values comparable to those of previous transient N‐MOSFETs) and using a Si NM and Mg electrode in the range of 350 to 660 cm^2^ s V^–1^ mobilities and 10^5^ on‐off ratio.^[^ ^9^, ^39^, ^40^, ^41^ ^]^ The transfer characteristics of the N‐MOSFET are shown in Figure S7, Supporting Information. Transient MOSFETs rapidly lose their electrical output in aqueous environment because of the fast hydrolysis of the conducting metal layer.^[^ ^9^ ^]^ Adding and/or stacking a biodegradable encapsulation layer, such as MgO, SiO_2,_ Si_3_N_4_, poly(lactic*‐co‐*glycolic acid) (PLGA), and silk, and adjusting the thickness of encapsulation, tune the diffusion rate of water molecules and delay circuit dissolution, thus extending the functional lifetime.^[^ ^9^, ^42^ ^]^ Transient devices with encapsulation show a two‐stage transition: the first stage of stable operation, with protection from water diffusion by the encapsulation layer, and a second stage of rapid transience arising from dissolution of the active component (usually metal electrode) when water penetrates the encapsulation layer.^[^ ^9^, ^39^, ^42^ ^]^ Figure 4c shows the two‐stage functional transiency of the N‐MOSFET with a MgZnCa MG electrode and a PBAT (100 µm thick) encapsulation layer in PBS at room temperature. The N‐MOSFET operates stably for 3 days and loses its original performance over the next 5 days. A transition in degradation rate was observed during the degradation stage, whereas the transiency of the MOSFET with the Mg electrode is usually very rapid without transition.^[^ ^9^, ^39^, ^40^ ^]^ The N‐MOSFET transition occurs in two steps because MgZnCa MG shows two different dissolution rates (nonlinear dissolution). In addition, the dissolution rate of MgZnCa MG is lower than that of Mg, so the performance drop rate in MgZnCa MG N‐MOSFET is slower than Mg. MgZnCa MG can be also available to the other passive and active components such as capacitors, inductors, and diodes. Figure S8, Supporting Information shows the optical images and electrical performances of inductors, capacitors, and diodes with comparable performance.

MgZnCa MG can also be used in various transient sensors. Figure 4d presents arrays of MgZnCa MG temperature sensors using the thermal resistivity of the material. Figure S9, Supporting Information shows the negative temperature coefficient of a MgZnCa MG resistor with a value of 0.3 Ω °C^–1^ in the temperature range of 25 to 40 °C. An array of MgZnCa MG temperature sensors provides comparable measurements of temperature variations of a joule‐heated rod in infrared camera measurements (Figure 4e). The large stretchability and the fatigue resistance of MgZnCa MG make it easy to integrate with mechanical energy harvesters, especially those requiring large repeatable mechanical deformation. Figure **4f**  shows exploded view of a transient TENG (left) made of triboelectric layers (PBAT and PCL, ≈100 µm thick) with a microstructure (right) to widen the contact area, a PLGA spacer (≈80 µm thick) for electrical separation, and MgZnCa MG electrodes for electron collection (≈100 nm thick) (see details in the Experimental Section) as an example of mechanical energy harvest. The open‐circuit voltage (*V*_oc_) and short‐circuit current (*I*_sc_) of a transient TENG are ≈20 V and ≈1.3 µA (Figure S10, Supporting Information). Figure 4g shows the durability of the transient TENG with MgZnCa MG under repeated stretching compared to the conventional Mg electrode. The transient TENG with the MgZnCa MG electrode maintains its initial peak‐to‐peak voltage (*V*_pp_initial_) even after 50 000 fatigue bending cycles at 1.5% strain owing to the high yield strain of the MgZnCa MG electrode, while the transient TENG with the Mg electrode loses up to ≈60% of *V*_pp_initial_ (Figure 4g). Figure 4h shows a series of dissolution images of the transient TENG with the MgZnCa MG electrode in PBS (pH 7.4) for 48 h at room temperature. The MgZnCa MG electrode partially dissolved after 12 h, and fully dissolved after 48 h apparently. Biodegradation time of polymeric layers of TENG (PLGA, PCL, and PBAT) is reported to take various time extents; weeks (4 weeks; PLGA 50/50 in PBS at 37℃ and 11–12 weeks; PBAT in mature compost at 58 °C), and years (>2 years; PCL).^[^ ^43^, ^44^, ^45^ ^]^

### 2.4 In Vitro and In Vivo Biocompatibility

**Figure 5:**
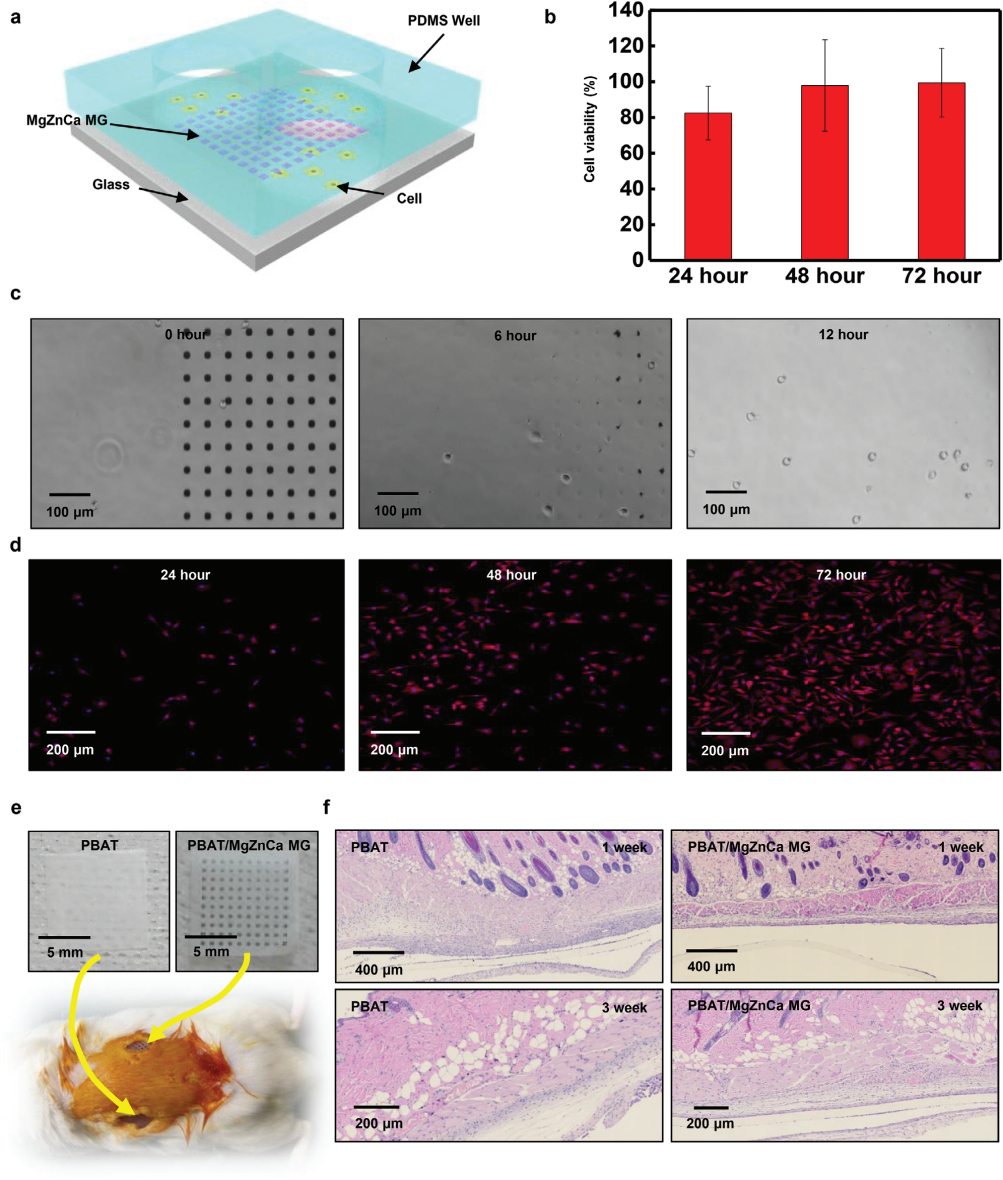
In‐vitro and In‐vivo biocompatibility of Mg_67_Zn_28_Ca_5_ MG. a) Schematic image of test platform for in‐vitro cell viability test. L929 cells are cultured on a glass substrate, half of which is a dot‐patterned MgZnCa MG array (10 µm × 10 µm × 500 nm); the other half is bare glass surface for better initial adhesion for cell in a custom‐made PDMS chamber (8 mm diameter). b) L929 cell viability for 72 h. Cell viability is calculated from the number of cells attached to bare slide glass (control) and the number of cell attached to MgZnCa MG. c) Image of cell attachment during MgZnCa MG dot‐array dissolution in cell medium (RPMI 1640) over 12 h. As MgZnCa MG pattern dissolves, the suspended cells in the cell medium spontaneously attached to the surface of the patterned area as well as the bare surface of the slide glass. d) Fluorescence image showing cell proliferation after MgZnCa MG dissolution for 72 h. e) Image of implanted PBAT/MgZnCa MG and PBAT sample in the back of the ICR mouse. f) Images of H&E stained skin sections at 1 and 3 weeks, respectively, after PBAT/MgZnCa MG and PBAT implatation.

The biocompatibility of MgZnCa MG is important for biomedical electronic applications. Biocompatibility of bulk MgZnCa MG with various compositions has been reported in some studies.^[^ ^31^, ^46^, ^47^ ^]^ For example, cell tests using L929 and MG63 on Mg_66_Zn_30_Ca_4_ and Mg_70_Zn_25_Ca_5_ bulk MG shows good biocompatibility.^[^ ^46^ ^]^ Mg_60_Zn_35_Ca_5_ shows good biocompatibility in the indirect MTT assay of MC3T3 on a cold isostatic press (CIP) and in vivo inflammation tests.^[^ ^31^, ^47^ ^]^ However, the biocompatibility of electronic‐grade MgZnCa MG has not been investigated still. **Figure** **5** summarizes the in vitro and in vivo toxicity tests for electronic‐grade nanoscale Mg_67_Zn_28_Ca_5_ MG.

Figure 5a–d shows the cytotoxicity test using the viability of L929 cells. Figure 5a demonstrates the cell culture platform for verifying the biocompatibility of the MgZnCa MG. Half of the cell culture area of the platform is MgZnCa MG patterned and the other half is a bare glass substrate. Cell viability was assessed through the attachment rate of L929 cells to the MgZnCa MG patterned substrate. The relative cell viability of MgZnCa MG compared to the control group was over 80% after 24 h, and over 97% after 48 h, indicating no toxicity of the substrate to the cells (Figure 5b). There was relatively a lower viability (≈80%) at 24 h compared to the control group (≈100%). The physical instability of MgZnCa MG patterned area during the corrosion may affect cell attachment and caused the viability at 24 h slightly underestimated, but after completion of dissolution (after 48 h), the average cell viability increased to 97.5%. Figure 5c,d shows the optical image of cell spreading within 12 h and fluorescence image of cell proliferation after 72 h, respectively. A few cells were observed just after cell culture on the MgZnCa MG (0 h, Figure 5c). The patterned MgZnCa MG gradually dissolves and the cells start to attach to the patterned area (6 h and 12 h at Figure 5c). After complete spreading (for 24 h),^[^ ^48^ ^]^ a proper proliferation of the cells was observed for 72 h (Figure 5d and Figure S11, Supporting Information). Figure 5e and Figure S12, Supporting Information shows an MgZnCa MG (300 nm thick)/PBAT (40 µm thick) and PBAT (40 µm thick) implanted in the back of a mouse. Visual inspection of the implantation sites revealed no sign of inflammation. Hematoxylin and eosin (H&E) staining of skin sections demonstrated comparable levels of immune cells, including polymorphonuclear cells, lymphocytes, and plasma cells compared to the PBAT control groups (Figures 5f). Taken together, these results suggest that the MgZnCa MG/PBAT examined here is biocompatible and has the potential to be used for long‐term implantation of months to years.

## 3 Conclusion

Biodegradable MgZnCa MG nano‐films offer greatly improved stretchability and fatigue resistance with high yield strain and elongation compared to biodegradable crystalline metals. Integrability with other biodegradable electronic materials provides a wide range of applications in stretchable transient devices. The demonstration of a fatigue‐resistant suggests the promising use of nanoscale MG in mechanical energy harvesters. The biocompatibility of MgZnCa MG confirms its extensibility for biomedical applications such as stretchable sensors and energy sources.

## 4 Experimental Section

##### Formation and Characterization of Electronic Grade MgZnCa MG Films

Magnetron co‐sputtering from separate targets Mg, Zn, and Ca (99.99% purity, Mg and Zn from iNexus, Inc., Korea, and Ca from VTM, Korea) at a base pressure of 1 × 10^–6^ Torr and a working pressure of 5 mTorr in Ar gas at room temperature yielded a thin Mg_67_Zn_28_Ca_5_ MG film (≈300 nm thick) on Si. The average composition of the MgZnCa MG film was confirmed by EDS. XPS (K‐Alpha system, ThermoFisher Scientific, USA) provided an in‐depth composition of Mg_67_Zn_28_Ca_5,_ while Ar ions etched the surface of the MG film. The relative amount of metal products were calculated using deconvolution of obtained XPS peaks based on their standard binding energy of metals and metal products. TEM (JEM‐2100, JEOL) and FFT pattern images at an accelerating voltage of 200 kV confirmed the amorphous nature of MgZnCa MG film. A serpentine trace of thin Mg_67_Zn_28_Ca_5_ MG resistor (≈300 nm thick) was formed by sputtering using a shadow mask (stainless steel, 500 µm thick).

##### Dissolution Chemistry of MgZnCa MG

Patterned MgZnCa MG (300 nm thick) on SiO_2_/Si wafer immersed in PBS (Sigma Aldrich, cat. no. 806552) at pH 7.4, BS (boric acid/potassium chloride/sodium hydroxide) with pH 9.0 (Merck), and DI water at pH 7.0 (Daejung Chemical & Metals, Korea) at 37 °C. Samples were collected at various dissolution times, rinsed in DI water, dried, and used for the following measurements. Two‐point resistance was measured using a digital multimeter with Au pads on each side of the resistor to minimize contact resistance. XPS was used to observe the surface chemistry with variations in the binding energy during dissolution. Ar ions etched the surface of the MG film during XPS analysis in order to obtain an in‐depth profile of the chemical binding state during dissolution. Surface morphology was also observed by SEM.

##### In Situ Micro‐Tensile Testing

Freestanding 300‐nm‐thick MgZnCa MG, Mg, and CuZr MG thin films were prepared by undercutting the Si substrate using XeF_2_ gas. Dog‐bone‐shaped tensile specimens with 8 µm gauge length and 4 µm width were machined by focused ion beam milling (Quanta 3D FEG, FEI, USA) and transferred onto push‐to‐pull devices (Hysitron) using an omniprobe system. In situ tensile tests were conducted using an in situ nanoindenter (PI‐87 Picoindenter, Hysitron, USA) with a diamond flat punch tip.

##### Fabrication of a Transient Electronic Device with a MgZnCa MG Electrode

Transient passive devices (stretchable electrode and temperature sensors) were fabricated by preparing a polybutylene adipate threphtalate (PBAT, S‐EnPol, Korea) substrate by solvent‐casting (1 g) PBAT pellets in (10 mL) of chloroform on glass. MgZnCa MG and Mg were sputtered directly on a PBAT substrate. Patterning with laser ablation (MD‐U1000C, Keyence, Japan) formed fine serepentine features to interconnect the electrode and the temperature sensor array. A commercial blue LED (3.2 mm wide × 1.6 mm long, Rohm semiconductor, Japan) was attached to contact pad by conductive epoxy (MG chemicals, Canada) by heating to 60 °C for 2 h. For TENGs, polycarprolactone (PCL, Mn ≈ 80 000, ≈100 µm thick, Sigma Aldrich, USA) and PBAT (≈100 µm) were solvent‐cast on a photolithographically patterned silicon mold to form micropillars. An electrode layer of MgZnCa MG was sputtered on the flat side of the PCL and PBAT triboelectric layers for ≈100 nm thickness. Poly(lactic*‐co‐*glycolic acid) (PLGA, 65:35, ≈80 µm thick, Sigma Aldrich, USA) for spacers was prepared by solvent casting and patterned by laser cutting. The PCL and PBAT triboelectric layers with the MgZnCa MG electrode were peeled from the silicon mold and integrated with the PLGA spacer by heat bonding. Fabrication of transient N‐MOSFETs began with phosphorus doping on silicon‐on‐insulator (top silicon ≈400 nm, SOITEC, France) at 1050 °C for 5 min. By patterning a 3 µm × 3 µm hole, undercutting buried oxide in hydrofluolic acid (HF, 49%, Transene Company, USA) for 30 min, transferring n‐doped Si NM onto a poly (methylmethacrylate)/diluted polyimide coated glass substrate and isolating the doped area by reactive ion ethcing (RIE, J Vacuum Technology, Korea) formed an n‐channel on the sacrificial layer. Depositing a thin layer of SiO_2_ (≈100 nm) by PECVD and patterning it by wet etching of buffered oxide ethchant (BOE, 6:1, Transene Company, USA) formed the gate dielectric. Lifting off the sputtered 300 nm thick MgZnCa MG defined the gate, source, and drain electrodes.

##### Characterization of Stretchability and Fatigue Behavior

A tensile sample was stretched in a custom stretching jig. Fatigue testing of MgZnCa MG, Mg, and CuZr MG was conducted using a multi‐mode fatigue tester (CKMF‐12P, CKSI, Korea) at the speed of 1 Hz per cycle. The change in the electrical resistance of MgZnCa MG, Mg, and CuZr MG electrodes during fatigue testing was recorded using a digital multimeter (NI USB‐4065, National Instruments, USA). The *V*_pp_ of the TENG was measured using an oscilloscope (TBS1052B, Tektronix, USA) after every 5000 cycles of fatigue bending.

##### In Vitro Cell Viability Test

MgZnCa MG dot arrays (10 µm wide, × 10 µm long, × 500 nm thick) were fabricated on a glass substrate via a liftoff process with AZ nLOF2070. A PDMS chamber (10:1, Dow Corning, USA) was made by puncturing an 8‐mm‐diameter hole on a 5‐mm thick PDMS piece. The bottom of the PDMS chamber was treated with an ultraviolet ozone for proper attachment to the glass substrate. L929 cells were thawed and maintained in RPMI 1640 medium (Gibco, MA, USA) containing 10% fetal bovine serum (FBS, VWR International, PA, USA) and 1% penicillin/streptomycin (Gibco). In each PDMS chamber, 2000 cells were seeded. The phase contrast microscopic images of the cells were taken at every time point (Eclipse TS100, Nikon Instruments, Japan) after cell seeding. Also, at each time point, 4% paraformaldehyde (Bylabs, Korea) was used to fix the cells. The cells were penetrated with 0.25% Triton X‐100 (Sigma Aldrich) in PBS, followed by blocking with 2% FBS and 0.5% tween 20 (Sigma Aldrich) in PBS. Then, the DNA and F‐actin of the cells were stained with conjugated fluorescence marker for an hour in 37 °C (Hoechst 33342, Invitrogen; 1:1000, Alexa Fluor 594 phalloidin, Invitrogen; 1:200, respectively). Fluorescence images were obtained using a confocal laser scanning microscopy (LSM 700, Carl Zeiss, Germany). The number of DNA in every image was counted manually to figure out the number of cells attached on the surface of the MG.

##### In Vivo Evaluation Test

MgZnCa MG (≈300 nm) was sputtered onto a PBAT substrate (≈40 µm) through a shadow mask made from the Kapton film (≈25 µm) with the help of laser cutting. All the experiments on animals were performed in accordance with the national and institutional guidelines and the Guide for the Care and Use Committees of laboratory animals based on protocols approved by the Korea University (KUIACUC‐2013‐93, IRB number: KOREA‐2020‐0060). Twelve Institute of Cancer Research (ICR) mice were anesthetized via inhalation (1 L min^–1^ of gas mixed with 50–70% medical oxygen and 2% isoflurane). After shaving the center of the mouse and making an incision, the skin was peeled off. PBAT/MgZnCa MG and PBAT were implanted on the lower left and right parts of the skin to the lower layer of the skin then observed for 3 weeks. The tissue samples were fixed in 10% neutral buffered formalin, embedded in paraffin, sliced to 4 µm thickness, and stained with hematoxylin and eosin (H&E staining). Tissue images were taken on a Leica M165 FC stereomicroscope equipped with a LEICA DFC310FX camera using the Leica application suite version 3.4.1 software program.

## Conflict of Interest

The authors declare no conflict of interest.

## Supporting information

Supporting Information

Supplemental Video 1

## Data Availability

Research data are not shared.
