# Supplementary material for: Biodegradable Metallic Glass for Stretchable Transient Electronics
Source: Adv Sci (Weinh). 2021 Mar 15;8(10):2004029. doi: 10.1002/advs.202004029 (PMC8132068; doi:10.1002/advs.202004029)
Supplement: Supplementary file 1 — Supporting Information [file ADVS-8-2004029-s001.pdf]

WILEY-VCH

© 2021 Wiley-VCH GmbH

ADVANCED SCIENCE
Open Access

Supporting Information

for *Adv. Sci.,* DOI: 10.1002/advs.202004029

Biodegradable Metallic Glass for Stretchable Transient Electronics

Jae-Young Bae, Eun-Ji Gwak, Gyeong-Seok Hwang, Hae Won Hwang, Dong-Ju Lee, Jong-Sung Lee, Young-Chang Joo, Jeong-Yun Sun, Sang Ho Jun, Myoung-Ryul Ok*, Ju-Young Kim*, and Seung-Kyun Kang**

WILEY-VCH

© 2021 Wiley-VCH GmbH

Supporting Information

Biodegradable Metallic Glass for Stretchable Transient Electronics

Jae-Young Bae, Eun-Ji Gwak, Gyeong-Seok Hwang, Hae Won Hwang, Dong-Ju Lee, Jong-Sung Lee, Young-Chang Joo, Jeong-Yun Sun, Sang Ho Jun, Myoung-Ryul Ok*, Ju-Young Kim*, and Seung-Kyun Kang**

Figure S1. Film composition measured using electron XPS in SEM. a) Peak identification, b) Table of atomic composition of the MgZnCa MG film.

a

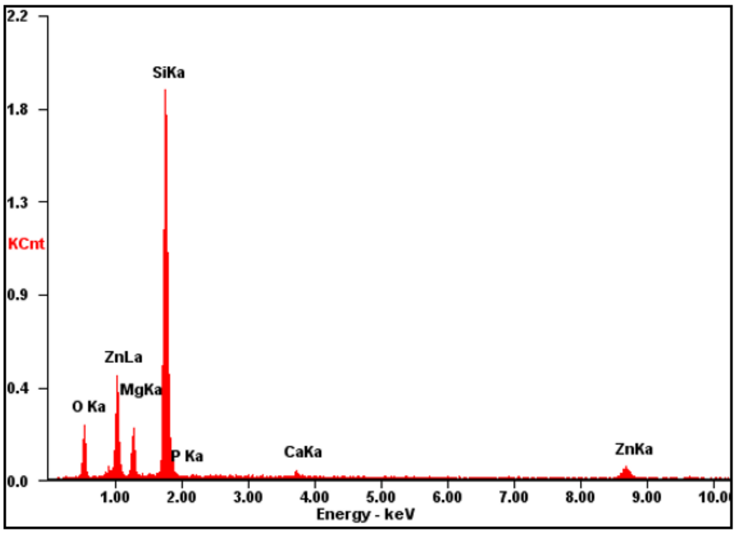

b

Element	at.%
Mg	11.0
Zn	4.6
Ca	0.8
O	29.6
Si	54.0

Figure S2. Dissolution image of MgZnCa MG serpentine electrode trace (1.5 mm width × 67.5 mm long × 300 nm thick) for 300 min.

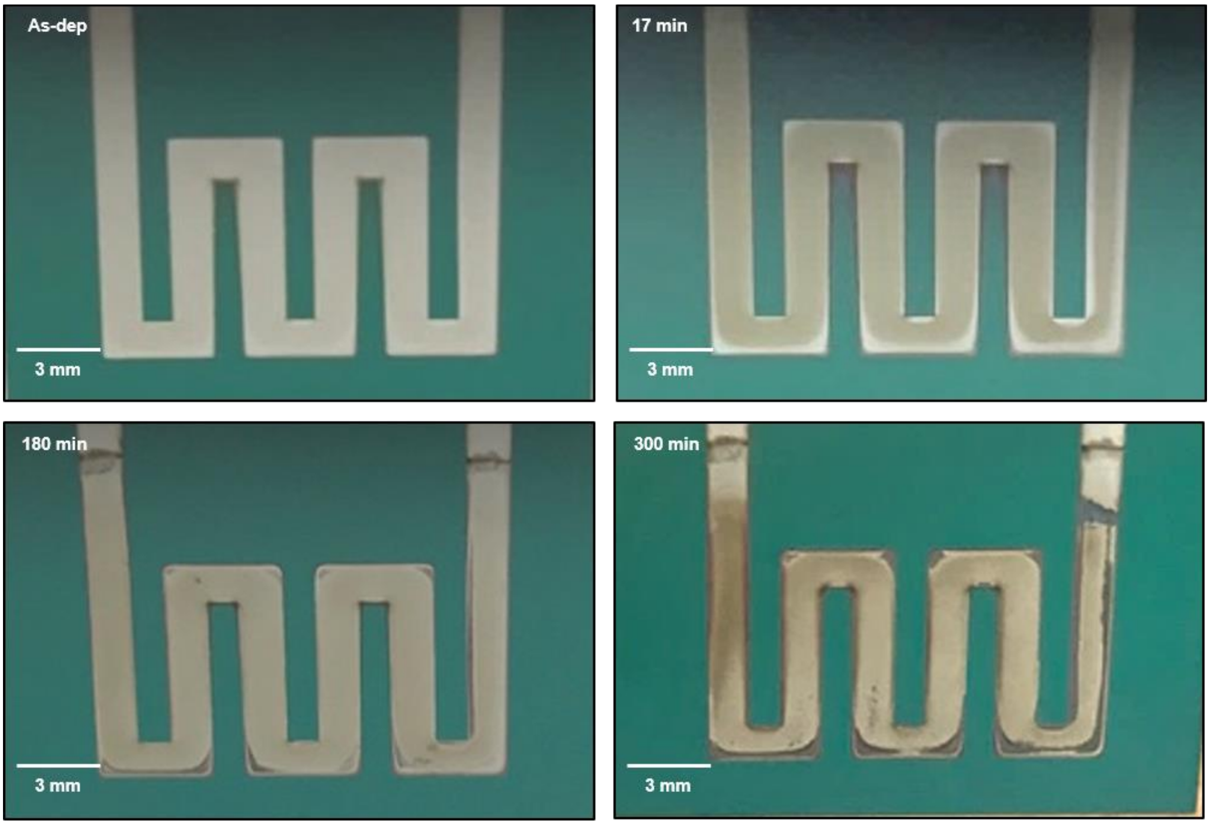

Figure S3. Surface morphology and EDS analysis of serpentine MgZnCa MG dissolved in PBS at 37°C for a) 17 min and; b) 180 min.

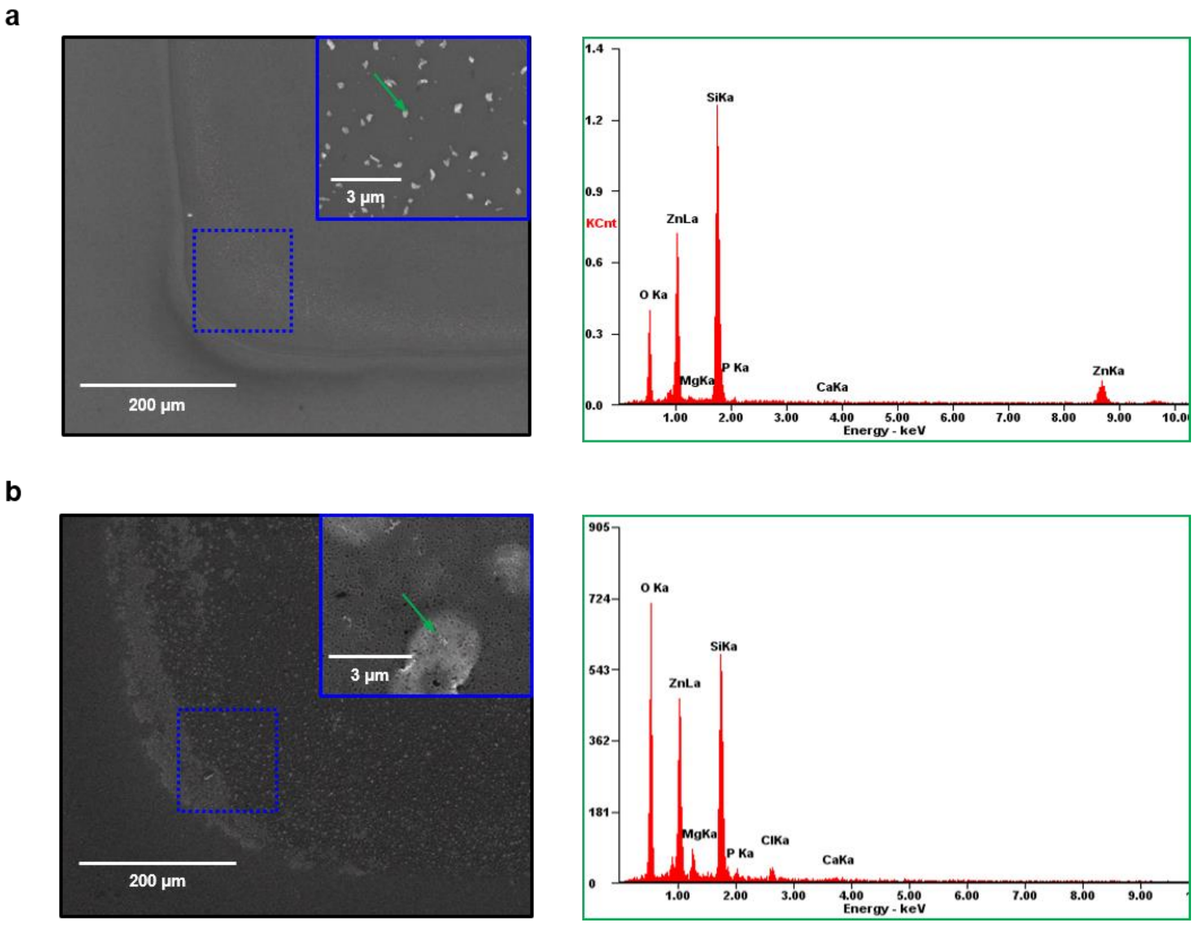

Figure S4. Material profile at various depths during dissolution in PBS (pH 7.4) after a) 17 min and b) 180 min.

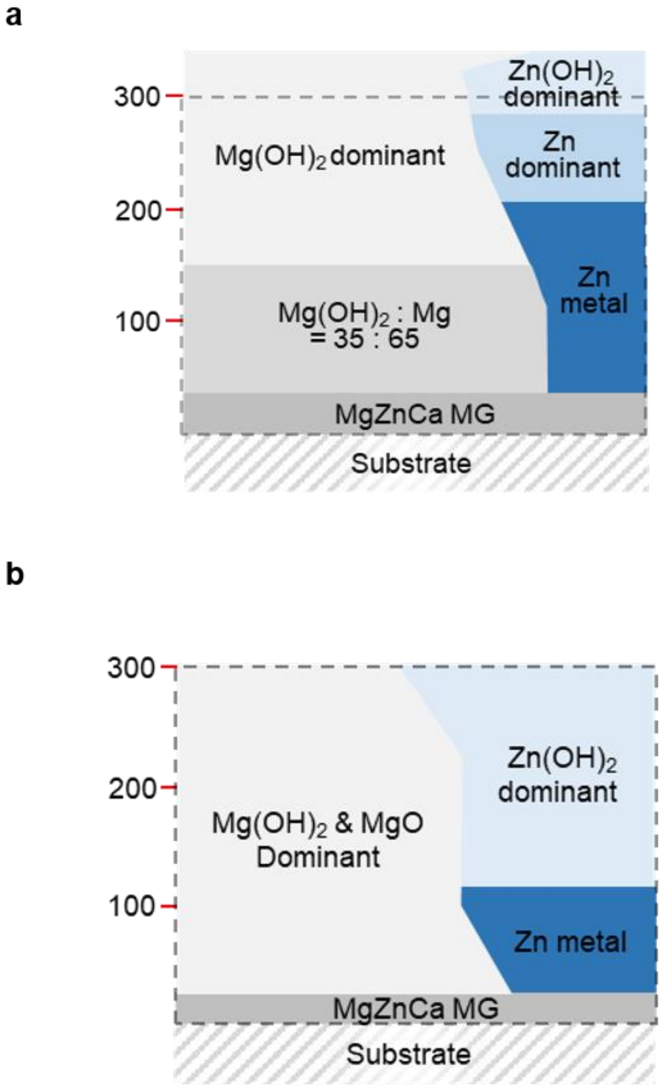

Figure S5. a) FEM simulation of dog bone shape MgZnCa MG (300 nm) on a PBAT substrate (150 μm) at 2% applied strain. b) Maximum strain versus applied strain on dog bone shape MgZnCa MG.

a

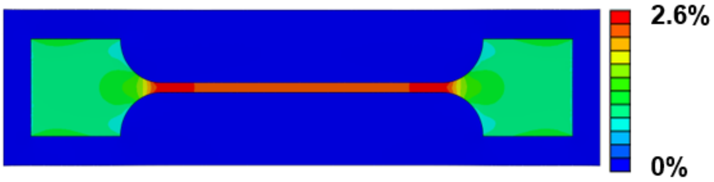

b

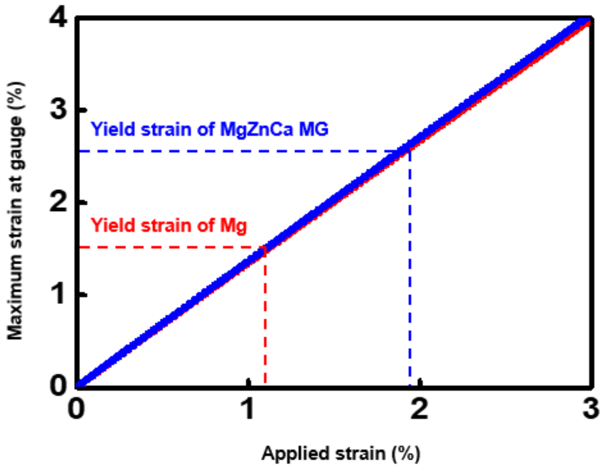

Figure S6. SEM image of a) Mg electrode after 10000 cycle at 2 % strain and b) Mg serpentine structure after 10 000 cycle at 100% strain.

a

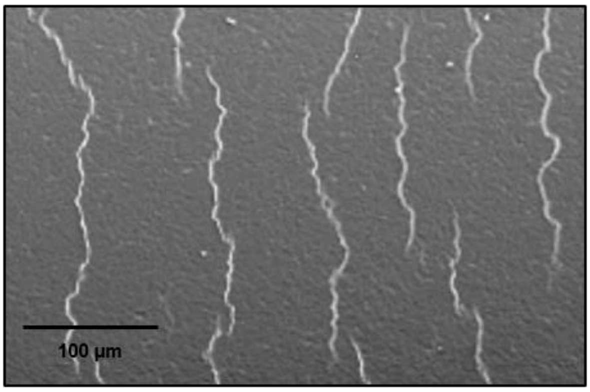

b

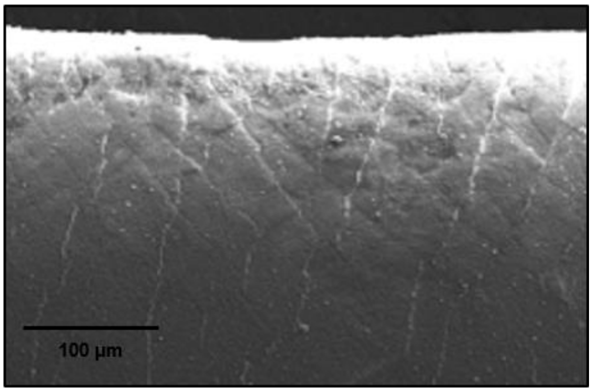

Figure S7. Linear (red) and log scale (blue) of the transfer characteristics of N-MOSFET.

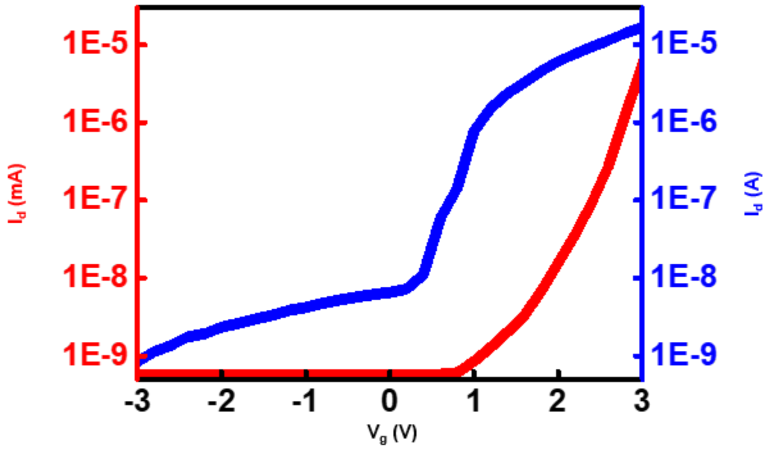

Figure S8. Passive and active components consisted of MgZnCa MG. a) Optical images of inductors (left) of various sizes and their inductances (right). b) Optical images of capacitors (left) of various sizes and their capacitances (right). c) Optical image of diode (left), I-V characteristics (middle), and its temperature coefficient (right).

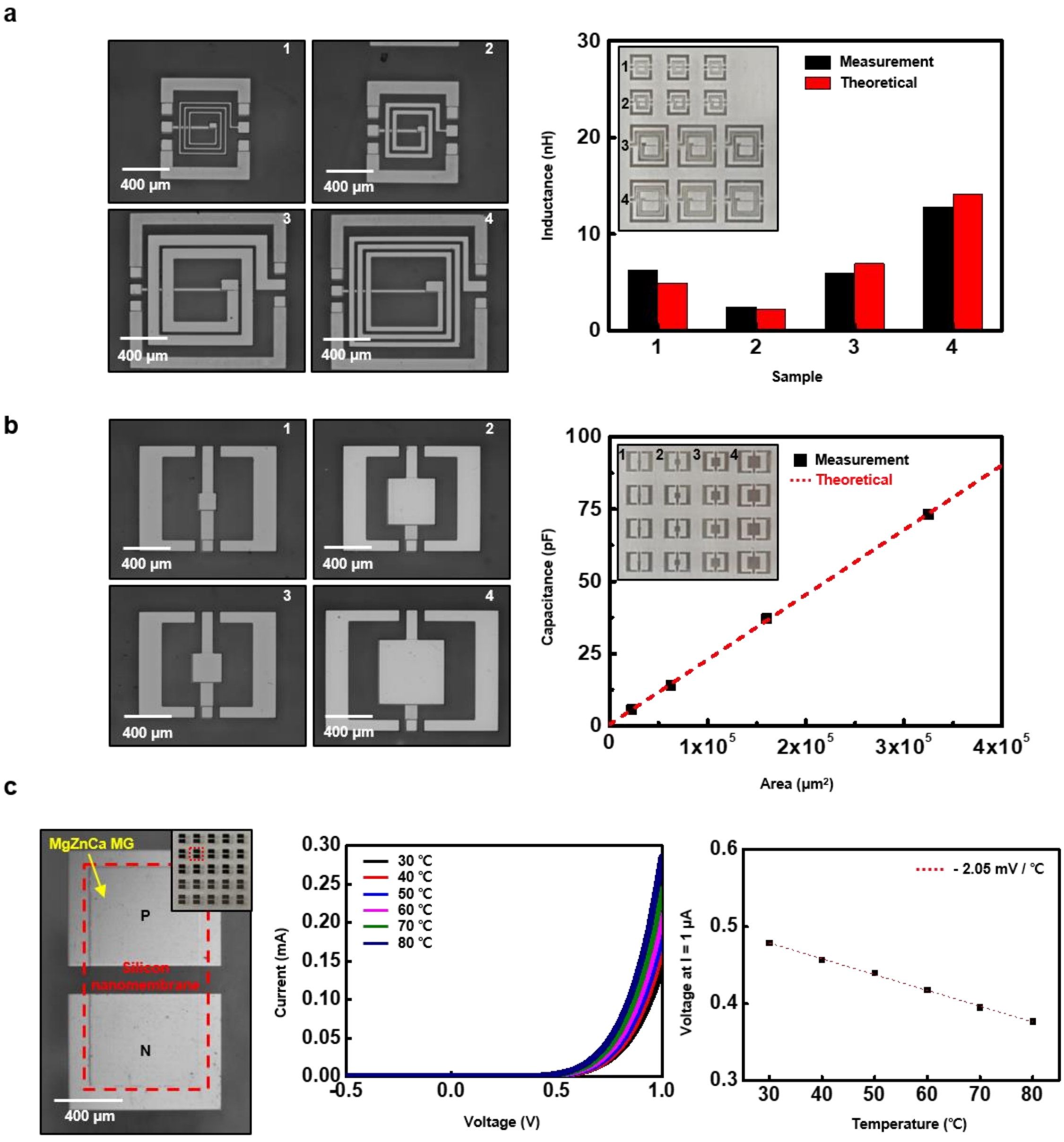

Figure S9. Resistance variation of the MgZnCa MG resistor (inset) with temperature.

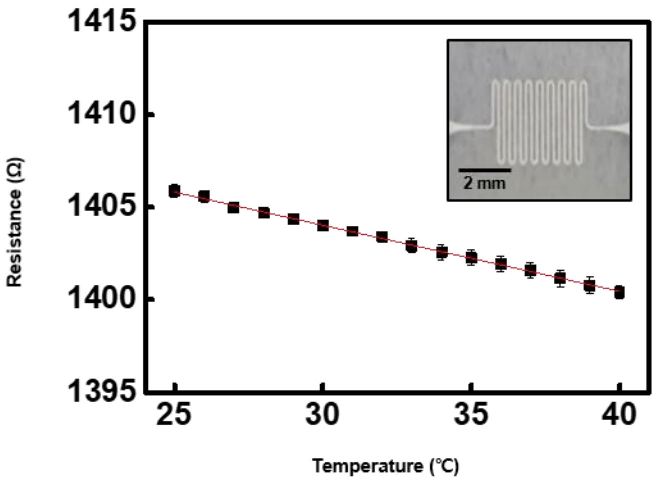

Figure S10. Electrical characteristics of MgZnCa MG TENG. a) Open circuit voltage (V_{oc}) and b) Short circuit current (I_{sc}) of biodegradable triboelectric nanogenerator.

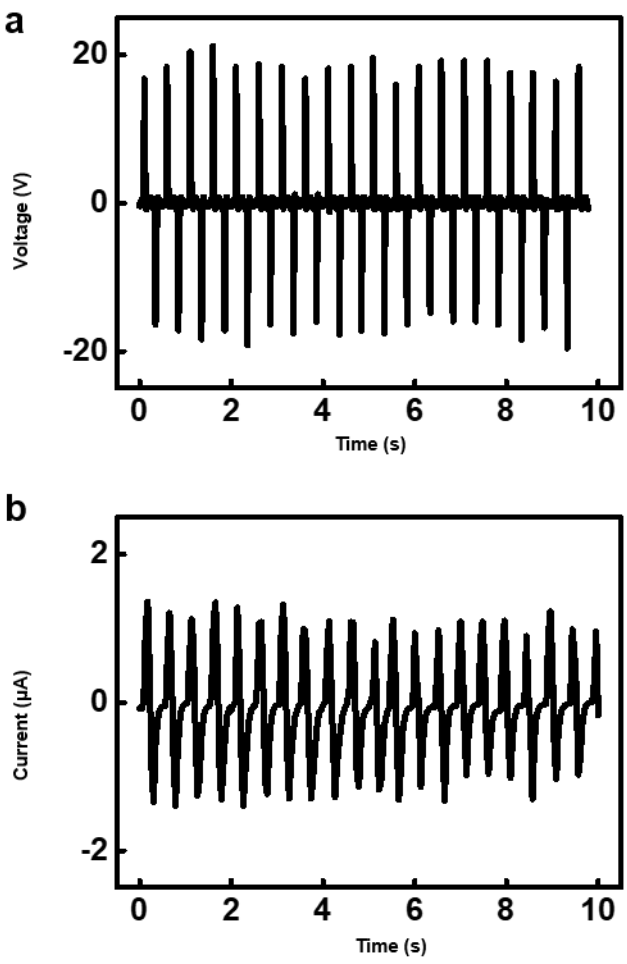

Figure S11. Optical image of in-vitro cell biocompatibility test over 3 days.

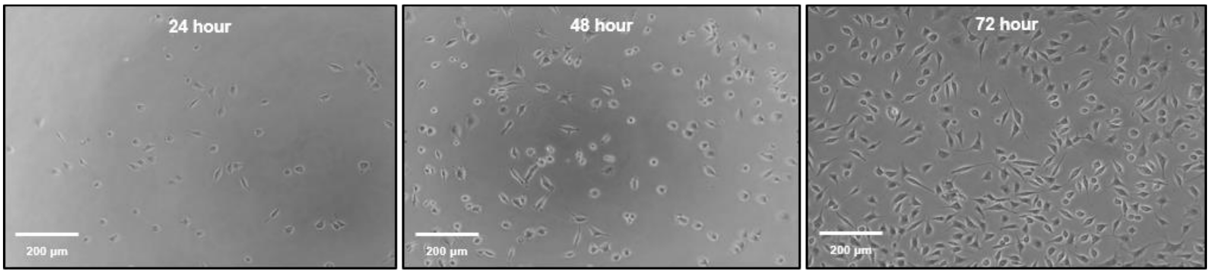

Figure S12. Magnified images of a) MgZnCa MG/PBAT and b) PBAT sample implanted in the back of the mouse

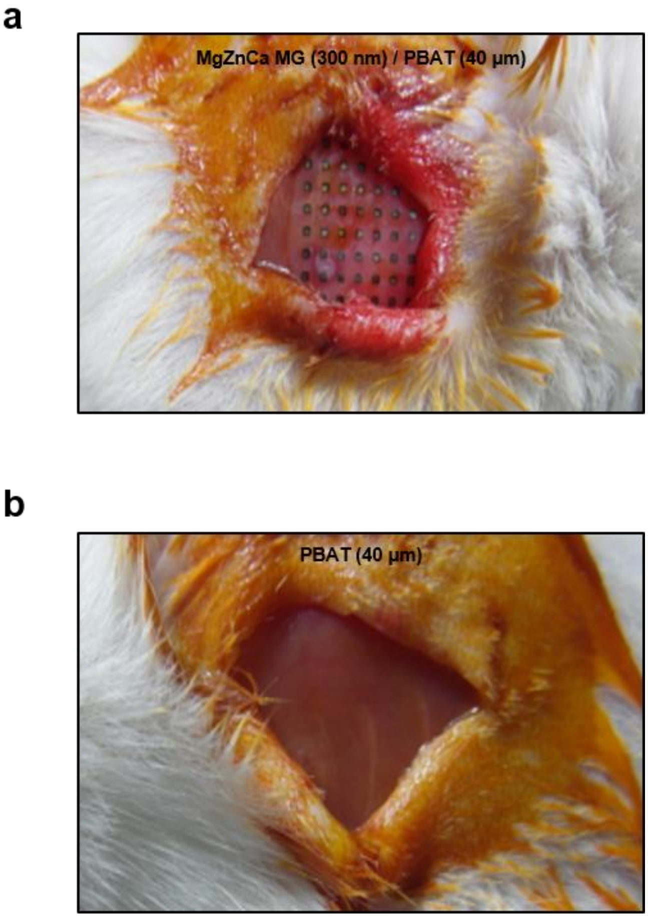

Supplementary video S1. In-situ tensile test of the nanoscale MgZnCa MG film (~300 nm).
